# The shapes of virulence to come

**DOI:** 10.1093/emph/eoy037

**Published:** 2019-01-12

**Authors:** Aakash Pandey, Daniel E Dawson

**Affiliations:** 1 Division of Biology, Kansas State University, Manhattan, KS, USA; 2 Department of Pathobiology and Population Health, College of Veterinary Medicine, North Carolina State University, Raleigh, NC, USA

## Definition and background

**Figure 1.**
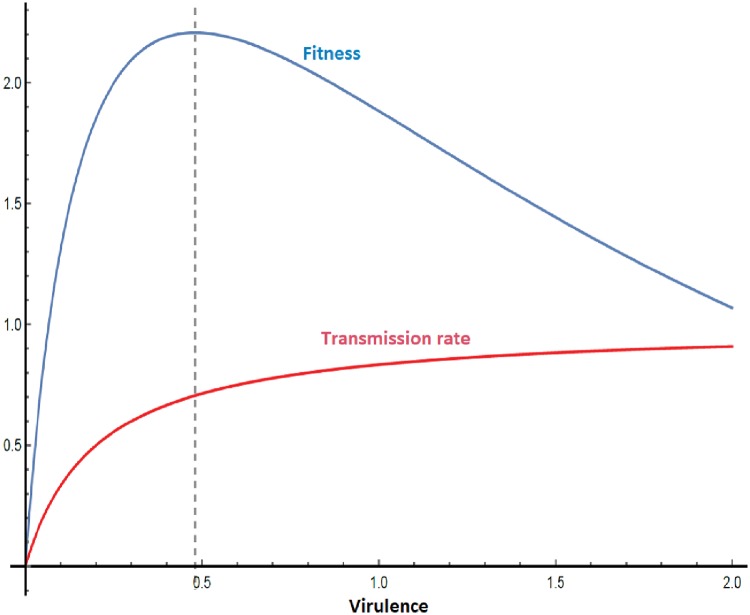
Classical model of virulence evolution operating under a virulence-transmission trade-off. Measured as the Basic Reproduction Number (R_0_), fitness is the product of transmission rate and duration of infection. Higher virulence increases the transmission rate (red line) but shortens the duration of infection, eventually reducing fitness. Given this trade-off, there is an optimal virulence corresponding to the maximum fitness (dashed line at the fitness curve peak). Since any mutants deviating from this intermediate virulence strategy have a lower fitness, we expect pathogens to evolve towards this intermediate evolutionary stable strategy

A pathogen’s virulence is the negative impact it has on a host. As a trait, virulence correlates with pathogen fitness, and hence can undergo selection. In directly transmitted pathogens, higher within-host growth rates are associated with higher transmission rates and greater fitness of the pathogen. Higher growth rates often result in higher virulence, resulting in the co-selection of virulence along with fitness. When expressed as mortality, increased virulence limits the amount of time an infected individual can transmit pathogens. This sets up a trade-off between infection duration and transmission probability, theoretically resulting in an optimal level of intermediate virulence [1] (Fig. 1). However, the nature of such trade-offs depends upon the life history strategies of both the pathogen and the host. Therefore, understanding virulence evolution pressures in different disease systems (e.g. vector-borne, environmentally transmitted and opportunistic) is critical to forecasting disease dynamics under changing conditions.

## Example in human biology and public health

Intermediate virulence may have evolved for HIV, as shown by Fraser *et al.* [2]. For this directly transmitted pathogen, set-point viral load (and hence growth that determines virulence) is clustered near the theoretical value that maximizes fitness (i.e. transmission potential). This level of virulence may represent an Evolutionary Stable Strategy (ESS) (Fig. 1), where mutants deviating from this virulence strategy have lower fitness and hence cannot invade the ESS [3].

## Example in clinical medicine

Anti-microbial resistance (AMR) often has fitness costs, and may influence virulence in bacteria [4]. Further, the evolution of virulence in the same species can be driven higher or lower by AMR depending upon the fitness balance of resistance to particular anti-microbial chemicals [5], as determined by interactions between AMR/virulence mechanisms, the environment, and the host [4]. This implies that AMR management could include anti-virulence strategies designed to minimize virulence through selection, potentially at the cost of increased AMR.
